# Osteoporosis is a neglected health priority in Arab World: a comparative bibliometric analysis

**DOI:** 10.1186/2193-1801-3-427

**Published:** 2014-08-12

**Authors:** Waleed M Sweileh, Samah W Al-Jabi, Sa’ed H Zyoud, Ansam F Sawalha, Mustafa A Ghanim

**Affiliations:** Department of Pharmacology/Toxicology, College of Medicine and Health Sciences, An-Najah National University, Nablus, Palestine; Department of Clinical and Community Pharmacy, College of Medicine and Health Sciences, An-Najah National University, Nablus, Palestine; Department of Biochemistry and Genetics, College of Medicine and Health Sciences, An-Najah National University, Nablus, Palestine

**Keywords:** Osteoporosis, Arab countries, Bibliometric, ISI, Web of Science

## Abstract

Osteoporosis is an important health problem with serious consequences. Evaluation of osteoporosis scientific output from Arab countries has not been explored and there are few internationally published reports on research activity about osteoporosis. The main objectives of this study were to analyze the research output originating from Arab countries and 3 Middle Eastern non-Arab countries, particularly Israel, Turkey and Iran in the field of osteoporosis. Original scientific articles or reviews published from the 21 Arab countries, Israel, Turkey and Iran about “osteoporosis” were screened using the ISI Web of Science database. The time frame for the result was up to year 2012. The total number of original and review research articles published globally about osteoporosis was 43,571. The leading country in osteoporosis research was United States of America (14,734; 33.82%). Worldwide, Turkey ranked 16th while Israel and Iran ranked 24th and 31st respectively. Among Arab countries, Egypt and Kingdom of Saudi Arabia came on positions 41 and 45 respectively. A total of 426 documents about “osteoporosis” were published from Arab countries which represents 0.98% of the global research output. Research about osteoporosis from Arab countries was very low until 2002 and then increased steadily. The total number of citations for osteoporosis documents from the Arab world was 5551 with an average citation of 13.03 per document and an *h-* index of 35. Thirty (7.04%) documents published from Arab countries about osteoporosis were published in *Saudi Medical Journal*. Egypt, with a total publication of 117 (27.47%) ranked first among the Arab countries in research about osteoporosis while American University in Beirut was the most productive institution with a total of 47 (11.03%) documents. Compared with other non-Arab countries in the Middle East, the research productivity from the Arab countries was lesser than that from Turkey and Israel but higher than that from Iran. The present data showed low research productivity in osteoporosis field in Arab countries. Research output can be improved by investing in more international and national collaborative research projects in the field of osteoporosis.

## Background

Osteoporosis is defined as a disease characterized by low bone mass and micro architectural deterioration of bone tissue, enhanced bone fragility and an increase in fracture risk (Kanis 1994; NIH Consensus Development Panel on Osteoporosis Prevention 2001). Unfortunately, the 2010 World Health Organization (WHO) Global Status report have discussed in detail the increased mortality and morbidity of many non-communicable diseases like cardiovascular, cancer and diabetes but osteoporosis was not mentioned in the report (WHO 2011). Approximately 1.6 million hip fractures occur worldwide each year and this number could triple or quadruple and reach between 4.5 and 6.3 million by the year 2050 which make osteoporosis a global disease (Roux et al. 2012). In Middle East, the problem of osteoporosis will soon be of greater importance considering the steady growth of the ageing population ((Maalouf et al. 2007); IOF 2011a, 2012). Iran accounts for 0.85% of the global burden of hip fractures and 12.4% of the burden of hip fractures in the Middle East (Soveid et al. 2005; Moayyeri et al. 2006; Ahmadi-Abhari et al. 2007; Beyranvand & Mohammadi 2009; Shahnazari et al. 2013). Furthermore, hypovitaminosis D is highly prevalent in Middle Eastern countries and might be a strong contributing factor for osteoporosis (Gannage-Yared et al. 2000).

The Middle East and Africa Audit about epidemiology, cost and burden of osteoporosis was published in 2011 by the International Osteoporosis Foundation (IOF). The audit focused on 17 countries including 11 Arab countries in the Middle East, Turkey, Iran and few other countries in Africa. According to the executive summary of the IOF audit report, osteoporosis is a neglected disease, not being integrated in medical curricula of most countries, and the level of awareness about osteoporosis is estimated as poor to medium in most studied countries (IOF 2011b). Furthermore, the audit stated that osteoporosis is not identified as a priority in most countries and that access to Dual-energy X-ray absorptiometry (DEXA) machines to measure bone mineral density is extremely limited. A primary recommendation of the IOF report is the need for more research to gather the necessary evidence that would aid health authorities to develop comprehensive healthcare policies at all levels for osteoporosis.

Bibliometrics refers to the implementation of statistical methods for evaluating the research productivity, for individuals, institutes and countries. Bibliometric analysis is a useful tool to obtain information about the current state of research in particular areas and allows researchers to identify new lines of research (Smith 2008; Smith 2012). Bibliometrics has been applied to various diseases and is now widely accepted as a method of measuring research and literacy output in any particular area ((Huber & Gullion 2003; Hofman et al. 2006; Smith 2010b; Smith 2010a; Rashidi et al. 2013; Bramness et al. 2014); Zyoud et al. 2014b; (Zyoud et al. 2014e)). Furthermore, the results of bibliometric analysis might help health policy makers and people in academia to shape up and direct research in the next decade. Bibliometrics of published research about osteoporosis from Arab countries has not been investigated before. Therefore, we conducted this study to analyze the quantity and quality of osteoporosis – based research productivity from Arab countries and compare it with that from non-Arab countries, particularly Turkey and Iran.

## Methods

The data used in this study were based on the ISI Web of Science, which is one of the world largest databases of peer-reviewed literature. The world-leading citation databases provide authoritative, multidisciplinary coverage from more than 12,000 high impact research journals worldwide (Thomson Reuters 2013). All Arab countries: Kingdom of Saudi Arabia (KSA); Egypt; Jordan; Lebanon; Qatar; Bahrain; Kuwait; Morocco; Tunisia; Syrian Arab Republic (SAR); United Arab Emirates (UAE); Iraq; Sudan; Yemen; Algeria; Comoros; Djibouti; Libya; Mauritania; Oman; Somalia, except Palestine, were used as country keys followed by “osteoporosis” key word as a search topic. Palestine was excluded from search keys because the Web of Science database does not recognize Palestine as an independent state yet. Because we are interested in osteoporosis and no other related terms, the key word used was restricted to osteoporosis. Furthermore, to increase the accuracy of results, research was refined and limited to original research articles and review articles because they represent the research activities, while other types of documents like editorials, conference proceedings, and others were excluded. The time frame for the result was up to year 2012. The 2013 and 2014 years were excluded because they are still open for new journal issues.

The database then generates a count of the total number of original articles, the total citations, and the value of the *h-*index (highly cited index). The *h-*index represents the number of citations received for each of the documents in descending order, while the *h-*graph measures the impact of a set of documents and displays the number of citations per document (for example: *h-*index of 10 means that there are 10 items that have 10 citations or more).The *h-*index was originally developed as a measure of qualifying research performance (Baldock et al. 2009; Schreiber 2013). Scientific output was evaluated based on a methodology developed and used in other bibliometric studies ((Sweileh et al. 2014b); Sweileh et al. 2014c; Zyoud et al. 2014b; Zyoud et al. 2014d; (Zyoud et al. 2014e)). The collected data were used to generate the following information: (a) total and trends of contributions to research about osteoporosis during all previous years up to December 31, 2012; (b) Arab countries research productivity and collaboration patterns; (c) journals in which Arab world researchers published; and (d) the citations received by the publications.

### Ethical approval

The Institutional Review Board (IRB) at An-Najah National University does not require submission of an IRB application for such study. The IRB considered that there is no risk for human subjects in such publications since the data are based on published literature and did not involve any interactions with human subjects.

### Statistical analysis

Data from ISI Web of Science were exported to Microsoft Office Excel® and then transferred to the Statistical Package for Social Sciences (SPSS; SPSS Inc., Chicago, IL, USA) program version 15 for analysis. The measurements of bibliometric analysis (e.g. countries, cited articles, institutions) were converted to the rank order using the standard competition ranking (SCR) (Zyoud et al. 2014c). We took into consideration the top 10 ranking in each item. If the measurements of bibliometric analysis have the same ranking number, then a gap is left in the following ranking numbers. The journal’s impact factors (IF) were evaluated using the Journal Citation Report (JCR; Web of Knowledge) 2012 science edition by Thomson Reuters (New York, NY, USA).

## Results

The total number of documents retrieved from ISI Web of Science using the methodology stated and without specifying the name of any country was 43,571. This number represents the global research productivity (original research articles and reviews) in osteoporosis as a research topic up to year 2012. Global research productivity in osteoporosis was much less than for hypertension (202,838 documents) and that for diabetes mellitus (126,187 documents). The leading countries in osteoporosis research were United States of America (USA) (14,734; 33.82%) followed by England (3,862; 8.86%) and Japan (3012; 6.91%). Worldwide, Turkey ranked 16th while Israel and Iran ranked 24th and 31 respectively. Among Arab countries, Egypt and KSA came on positions 41 and 45 respectively. At the global level, 40,811 (93.66%) of osteoporosis documents were written in English. The remaining documents were published by 24 different languages, mainly German (1,286; 2.95%) and French (634; 1.45%) languages. The annual global research productivity about osteoporosis remained low and steady until early 1990, after which, a steady and sharp increase in osteoporosis research was observed globally. The first article about osteoporosis was published in 1913 in by Conlon, FA at *Boston Medical and Surgical Journal.* The main research area of osteoporosis documents published globally was Endocrinology/ Metabolism (12754; 29.27%) followed by General/ Internal medicine (4,431; 10.17%). The journals in which most documents about osteoporosis were published include *Osteoporosis International (*2,685; 6.16%) followed by *Journal of Bone and Mineral Research* (1940; 4.45%) and *Bone* (1802; 4.14%). Approximately 5% (2,158) of global osteoporosis documents were open access. The most productive institutions of osteoporosis research were University of California at San Francisco (USA), Harvard University (USA), and University of Sheffield (UK) with a total of 948 (2.18%), 804 (1.85%) and 525 (1.20%) documents respectively.

**Figure 1:**
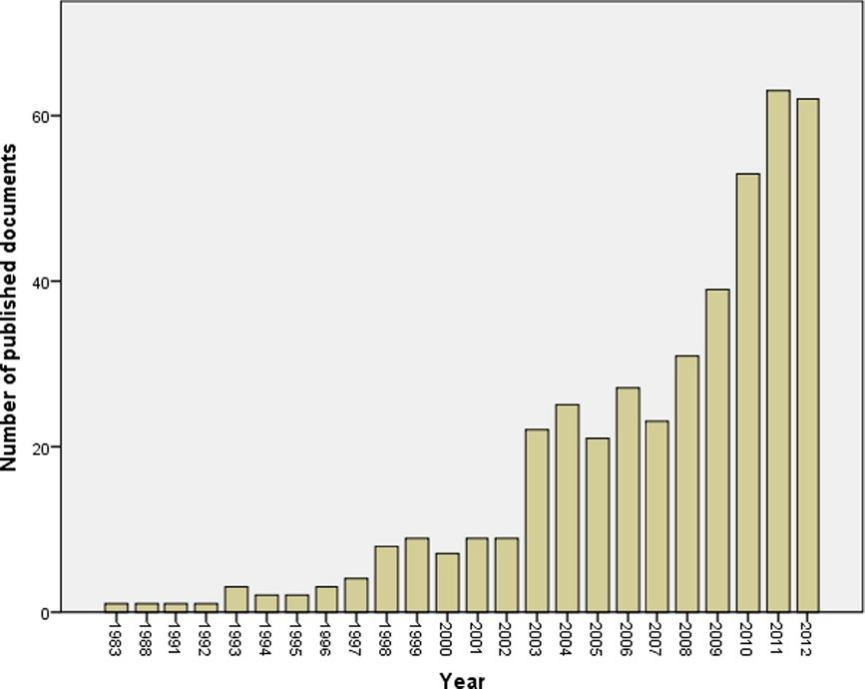
**Growth of osteoporosis research in Arab countries as extracted from ISA web of science using osteoporosis as a key topic search.**

When the same methodology was applied using the list of the 21 Arab countries, 426 documents were retrieved. Therefore, research about osteoporosis that was published from Arab countries represents approximately 0.98% of the global research productivity related to osteoporosis. Of the 426 documents, 412 (96.71%) were written in English language while 13 (3.05%) documents were published in French language and one document (0.24%) was published in German language. Sixty four osteoporosis documents (15.02%) published from the Arab world were open access while the remaining (362; 84.98%) were not open access. The annual number of documents published from Arab countries indicated that research activity about osteoporosis remained low until 2002 and showed a steady and double increase after 2003 (Figure 1). More than one third (41.78%) of documents about osteoporosis from the Arab world were published in 2010, 2011 and 2012 (Figure 1). When retrieved data was analyzed based on country production, Egypt (117; 27.46%) had the highest research output followed by Kingdom of Saudi Arabia (KSA) (97; 22.77%) and Lebanon (76; 17.84%). More than two thirds (290; 68.08%) of osteoporosis research from Arab world came from three Arab countries, particularly Egypt, KSA and Lebanon. No data related to osteoporosis was found from Somalia, Djibouti, Mauritania and Comoros (Table 1). The first article about osteoporosis co-authored by an Arab researcher/institution was published in 1983 (Sakati & Nyhan 1983). Endocrinology/ Metabolism (96; 22.53%) was the main research area of the 426 published osteoporosis documents followed by General/Internal medicine (68; 15.96%) and Rheumatology (42; 9.86%). Top 10 research areas of osteoporosis documents from Arab countries are shown in Table 2. More than half (226; 53.05%) of the 426 published osteoporosis documents from the Arab countries were related to females/women. Collaboration between Arab countries and non-Arab countries in osteoporosis research and publication was evident. Countries whose researchers collaborated most with investigators in the Arab world include the USA; (51; 11.97%) followed by France (28; 6.57%) and Canada (19; 4.46%).

**Table 1:** **Contribution of each Arab country to the 426 published documents about osteoporosis**

Country	Number of documents N = 426 (%)*
Egypt	117 (27.47)
Kingdom of Saudi Arabia	97 (22.77)
Lebanon	76 (17.84)
Morocco	51 (11.97)
Kuwait	28 (6.57)
Tunisia	22 (5.16)
Jordan	16 (3.76)
United Arab Emirates	11 (2.58)
Oman	9 (2.11)
Qatar	8 (1.88)
Libya	4 (0.94)
Iraq	2 (0.47)
Sudan	2 (0.47)
Algeria	1 (0.24)
Bahrain	1 (0.24)
Syria	1 (0.24)
Yemen	0
Somalia	0
Comoros	0
Djibouti	0
Mauritania	0

*Total exceeds 100% because of overlap in some documents among more than one Arab country.

**Table 2:** **Research areas of the 426 documents about osteoporosis published from the 21 Arab countries**

SCR ^a^	Research area	N = 426	%
**1st**	Endocrinology Metabolism	96	22.54
**2nd**	General Internal Medicine	68	15.96
**3rd**	Rheumatology	42	9.86
**4th**	Obstetrics Gynecology	23	5.40
**5th**	Pharmacology Pharmacy	19	4.46
**6th**	Orthopedics	17	3.99
**7th**	Geriatrics Gerontology	14	3.29
**7th**	Research Experimental Medicine	14	3.29
**9th**	Genetics Heredity	13	3.05
**9th**	Public Environmental Occupational Health	13	3.05

Abbreviations: *SCR* Standard Competition Ranking.

^a^Equal research areas have the same ranking number and then a gap is left in the ranking numbers.

Table 3 lists the top 10 journals in which documents about osteoporosis were published from Arab countries. Thirty (7.04%) documents about osteoporosis published from Arab countries appeared in *Saudi Medical Journal* which is a medical journal with wide medical scope based in KSA. Of the top 10 journals, 6 were in the specific field of bone and rheumatology, 3 had general medical scope, one was in the field of women’s health, and none has an impact factor above 5. Furthermore, 2 journals were based and published from KSA. Interest of Saudi researchers in osteoporosis research is evident in top 10 Arabic institutions involved in osteoporosis research (Table 4). Three of the top 10 productive institutions were based in KSA while 4 were based in Egypt. Of interest, the top productive institution was American University in Beirut (47; 11.03%). Seven institutions in the top 10 list are academic institutions, 2 are hospitals and 1 is a national research centre in Egypt.

**Table 3:** **Top 10 journals in which documents about osteoporosis were published from the 21 Arab countries**

SCR	Journal	Number of documents N = 426 (100%)	IF ^a^
**1st**	*Saudi Medical Journal*	30 (7.04)	0.619
**2nd**	*Journal of Clinical Densitometry*	26 (6.10)	1.713
**3rd**	*Osteoporosis International*	17 (3.99)	4.039
**4th**	*Bone*	16 (3.76)	3.823
**5th**	*Annals of Saudi Medicine*	14 (3.29)	1.123
**6th**	*Clinical Rheumatology*	12 (2.82)	2.037
**7th**	*Rheumatology International*	10 (2.35)	2.214
**8th**	*Maturitas*	9 (2.11)	2.844
**9th**	*Archives of Medical Science*	7 (1.64)	1.067
**10th**	*Journal of Bone and Mineral Metabolism*	6 (1.41)	2.219

Abbreviations: *SCR* Standard Competition Ranking, *IF* impact factor.

^a^The impact factor was reported according to Institute for Scientific Information (ISI) journal citation reports (JCR) 2012.

**Table 4:** **Top 10 active institutions in osteoporosis research from Arab countries**

SCR ^a^	Institute	Number N = 426 (%)	Affiliation
**1st**	American University Beirut	47 (11.03)	Lebanon
**2nd**	King Saud University	30 (7.04)	KSA
**3rd**	Cairo University	28 (6.57)	Egypt
**4th**	Mil Hospital Mohammed V	27 (6.34)	Morocco
**5th**	Kuwait University	24 (5.63)	Kuwait
**5th**	National Research Center	24 (5.63)	Egypt
**7th**	Ain Shams University	17 (3.99)	Egypt
**8th**	King Faisal Specialist Hospital Research Centre	15 (3.52)	KSA
**9th**	Mansoura University	12 (2.82)	Egypt
**10th**	King Fahd University Hospital	11 (2.58)	KSA
**10th**	Saint Joseph University	11 (2.58)	Lebanon

Abbreviations: SCR = Standard Competition Ranking; KSA = Kingdom of Saudi Arabia.

^a^Equal institutions have the same ranking number, and then a gap is left in the ranking numbers.

The total number of citations for osteoporosis documents from the Arab world, at the time of data analysis (March 28, 2014), was 5551 with an average citation of 13.03 per document. The total number of citations excluding self-citation was 5019. Of the 426 documents considered for the *h*-index, 35 had been cited at least 35 times at the time of data analysis. Compared with other non-Arab countries in the Middle East, the research productivity from the Arab countries was lesser than that from Turkey (number of documents = 873; total citations = 7467; number of citations per document = 8.55; h index = 33) and Israel (number of documents = 486; total citations = 16591; number of citations per document = 34.14; h index = 57) but higher than that from Iran (number of documents =185; total citations = 1471; number of citations per document = 7.95; h index = 17). Number of documents and citation analysis of osteoporosis documents published from Arab countries, Turkey, Israel and Iran is shown in Table 5.

**Table 5:** **Osteoporosis research output from Arab countries compared with that from Turkey, Israel and Iran**

Data/Country	21 Arab countries	Turkey	Israel	Iran
Number of population in millions	500	60	8	80
Number of published research articles and review articles	426	873	486	185
Total number of citations	5551	7467	16591	1471
Average citation per document	13.03	8.55	34.14	7.95
*h*-index	35	33	57	17

## Discussion

Reducing osteoporosis related morbidity and mortality in Arab countries requires an understanding of how these various countries progress in osteoporosis scientific research. Such understanding is instrumental for the development of an effective plan for this disease. Identifying research output and research activity in this field is of great importance to public health in general and women’s health in particular. Cultural and educational barriers make women in Arab countries more vulnerable to osteoporosis. One mechanism in overcoming such barriers is to shed more light on quantity and quality of osteoporosis research in the Arab world.

Our study was limited to 426 documents extracted from ISI web of Science, and, therefore, cannot be generalized to the osteoporosis literature covered by other databases such as Scopus and Google Scholar. However, the study does give a clear picture about the characteristics of published osteoporosis documents from Arab countries compared with that from Turkey, Israel and Iran. Although the quality and quantity of research in a particular area might differ from one search engine to another, ISI search engine remains one of the best official available tools for analyzing and tracking citations, and comparing citations among different research groups and different institutions. To the best of authors’ knowledge, this is the first article to analyze and compare the quantity and quality of osteoporosis-based research from Arab and non-Arab countries. Research indicators showed that research output particularly from Arab countries and Iran in the field of osteoporosis was low. Assessing the quality of research output is not an easy task. However, it was interesting to assess the quality of osteoporosis research in these countries as measured by the *h*-index. Unfortunately, the *h*-index for osteoporosis research output from Arab countries and Iran was also low. This was not surprising given that publishing high quality research requires significant effort, funding, and collaboration with international scientists which seem lacking in the field of osteoporosis research. This is of great importance since high quality publications allow established researchers to be able to obtain further funding and for young researchers to be more competitive in career advancement (Dawson 2013). Our results indicated that more effort is needed to bridge the gap in osteoporosis-based research from Arab and non-Arab countries compared with developed countries. Furthermore, osteoporosis needs to be given priority similar to other non-communicable diseases like diabetes mellitus and hypertension.

Results showed that the majority of top 10 institutions in osteoporosis research were universities with medical schools that were successful in making their contributions visible through ISI-indexed journals. This may be attributed to the emphasis by universities for academic staff to publish in indexed journals with high impact factor. Research productivity reveals intellectual output by the institution and is useful to university administrators when evaluating performance of university faculties in the light of university ranking among various universities (Zainal & Zainab 2011; Sweileh et al. 2014e).

To the best of our knowledge, this study is the first of its kind to obtain initial data regarding the publication and citation productivity of Arab and non-Arab countries in the Middle East in the field of osteoporosis in the ISI database. Few articles were published about osteoporosis bibliometrics. One study was recently published from India and came to similar conclusions to our findings and another study was published from China 2 decades ago (Wei 1995; (Bhardwaj & Ram 2013)). Internationally, several articles have been published about bibliometrics in rheumatology; however none was published about bibliometrics of osteoporosis (Maese Manzano 2009; Cheng & Zhang 2013). Rheumatology bibliometric studies have also pointed that some research field in rheumatology like arthritis is lagging behind (Glazier et al. 2001). The main goal of our study is to direct attention and to open doors for a scientific discussion among professionals and academics about osteoporosis research. Academic institutions in the Arab world are advised to initiate osteoporosis research and to strengthen research collaboration with international researchers and institutions in which osteoporosis research has evolved. For future studies in this direction, it is recommended that similar quantitative and qualitative research analyses for other disciplines, based on the same methodology, should be carried out. Our study is not without limitations, most of which are the same as those of studies performed in other biomedical fields ((Sweileh et al. 2014a; Sweileh et al. 2014d), b; (Zyoud et al. 2014a; Zyoud et al. 2014f)). First of all, articles published in non-ISI journals were not included, although they might contribute to scientific productivity. Another limitation is that some international journals do not recognize countries like Palestine as a separate country and publications from Palestine may be affiliated with Israel as a country. Therefore, some publications from Palestine might have been missed from our analysis. Finally, it should be noted that research output for certain institutions could have been under-estimated because of writing their English names differently in different articles. Therefore, such authors might have 2 or more institute profiles in ISI Web of Science database because their names were written differently in different articles.

## Conclusions

The present data showed that Arab countries have relatively low research productivity in the field of osteoporosis. Research output can be improved by investing in more international and national collaborative research projects in the field of osteoporosis.

## Abbreviations

SPSS: Statistical Package for Social Sciences
ISI: Institute for Scientific Information
KSA: Kingdom of Saudi Arabia
UAE: United Arab Emirates
SAR: Syrian Arab Republic
USA: United States of America
WHO: World Health Organization
JCR: Journal Citation Report
IRB: Institutional Review Board
IOF: International Osteoporosis Foundation
SCR: Standard Competition Ranking
IFs: impact factors
DEXA: Dual-energy X-ray absorptiometry.
